# Intake of Seafood in the US Varies by Age, Income, and Education Level but Not by Race-Ethnicity

**DOI:** 10.3390/nu6126060

**Published:** 2014-12-22

**Authors:** Lisa Jahns, Susan K. Raatz, LuAnn K. Johnson, Sibylle Kranz, Jeffrey T. Silverstein, Matthew J. Picklo

**Affiliations:** 1 United States Department of Agriculture, Agricultural Research Service, Grand Forks Human Nutrition Research Center, 2420 2nd Avenue N., Grand Forks, ND, 58203, USA; E-Mails: susan.raatz@ars.usda.gov (S.K.R.); luann.johnson@ars.usda.gov (L.K.J.); matthew.picklo@ars.usda.gov (M.J.P.); 2 Department of Food Science and Nutrition, 225 Food Science and Nutrition, 1334 Eckles Avenue Street Paul, MN 55108, USA; 3 Centre for Exercise, Nutrition, and Health Sciences, School of Policy Studies, University of Bristol, 8 Priory Road, Bristol, BS8 1TZ, UK; E-Mail: sibylle.kranz@bristol.ac.uk; 4 United States Department of Agriculture, Agricultural Research Service, Office of National Programs, 5601 Sunnyside Avenue, Beltsville, MD 20705, USA; E-Mail: jeff.silverstein@ars.usda.gov

**Keywords:** seafood intake, fish, shellfish

## Abstract

Current US federal dietary guidance recommends regular consumption of seafood (fish + shellfish) to promote health; however, little is known about how well Americans meet the guideline, particularly population subgroups that may be at risk for inadequate intake. The purposes of this study were to describe the prevalence of seafood consumption and, among consumers, the amounts of seafood eaten by sex, age group, income and education level, and race-ethnicity. Data from 15,407 adults aged 19+ participating in the 2005–2010 National Health and Nutrition Examination Surveys were analyzed using methods to account for sporadic intake of seafood. Over 80% of Americans reported consuming any seafood over the past 30 days, 74% reported consuming fish, and 54% reported eating shellfish. The percentages varied by socio-demographic group. Younger age and lower income and education levels were associated with lower odds of being a seafood consumer (*p* < 0.0001). Among those who reported eating seafood, the average amount eaten of any seafood was 158.2 ± 5.6 g/week. Among seafood consumers, women and individuals of lower age and education levels consumed less seafood. Approximately 80%–90% of seafood consumers did not meet seafood recommendations when needs were estimated by energy requirements. A great deal of work remains to move Americans toward seafood consumption at current recommended levels.

## 1. Introduction

Seafood (fish + shellfish) is a nutrient-rich lean protein food and is a component of many healthy eating patterns [1,2,3,4]. Consumption of seafood, particularly fish, is associated with reduced risk of cardiovascular disease [5,6]. While benefits are often ascribed to the long chain *n*-3 fatty acids found in fatty fish, a recent review that examined the association between either fish consumption or *n*-3 fatty acids and cerebrovascular disease concluded that the beneficial effects of fish consumption may be due in part to the contribution of other nutrients found in fish. Alternatively, seafood may replace other, less advantageous, protein foods in the diet [7]. The US Department of Agriculture (USDA) and US Department of Health and Human Services’ *Dietary Guidelines for Americans, 2010* [1] specifies consumption of at least 8 oz (227 g) seafood/week by all Americans two years old and older. Depending upon an individual’s energy needs, the recommended intake increases, and seafood should provide approximately one-fifth of the protein food group intake recommendation. No further information as to type of seafood recommended is specified; however, the recommendation encourages consumption of sources with high *n*-3 fatty acid content. 

Previous studies using self-reported dietary intake estimated that 70%–80% of individuals in the USA consumed seafood in the past 30 days, with a usual per capita intake of ~3.5–4.3 oz (99–122 g) seafood/week [8,9,10,11,12]. These estimates are similar to those derived from food disappearance data, which indicate that intake is approximately 4.4 oz (125 g)/week [13]. Age, income, and education have been found to be associated with fish intake in Europe [14,15]. A recent review of issues associated with fish consumption for cardiovascular risk reduction [6] indicated that information regarding seafood intake, particularly with respect to variability of consumption between population subgroups in the United States, was lacking. It is surprising, given that the US is a major consumer of seafood, and the known health benefits of seafood consumption, that we lack detailed knowledge of seafood intake by population subgroups in the USA [13,16].

The purposes of this study were (1) to describe the proportion of US adults who eat any seafood, fish, or shellfish, and (2) to estimate seafood, fish and shellfish intake amounts among adults who reported eating any seafood by sex, age, income, education, and race-ethnicity. We also evaluated how the amounts consumed compare to recommended intake levels. This information will be useful for targeting behavioral interventions to adults in efforts to improve compliance with the current recommendation for seafood intake. 

## 2. Methods 

### 2.1. Survey Design and Data

These analyses used data from the Centers for Disease Control and Prevention, National Center for Health Statistics (NCHS), National Health and Nutrition Examination Survey (NHANES) and the USDA, Agricultural Research Service (ARS), What We Eat in America (WWEIA) survey, the dietary assessment survey conducted in conjunction with NHANES. NHANES is a continuous cross-sectional survey of the civilian, non-institutionalized US population and includes detailed medical examinations and socio-demographic and health behavior questionnaires. The survey uses a complex, multistage probability sampling design with oversampling of selected socio-demographic subgroups of public health interest. Sample weights are provided to produce nationally representative estimates. Data are released in 2-year cycles and details may be found elsewhere [17]. NHANES protocols were approved by the NCHS Ethics Review Board and all participants provided informed consent. Pooled data from WWEIA, NHANES adult participants from 2005–2006 (*n* = 4652) [18], 2007–2008 (*n* = 5205) [19], and 2009–2010 (*n* = 5550) [20] were used for these analyses.

#### 2.1.1. Amount of Seafood Consumed

The WWEIA dietary intake survey consists of two non-consecutive, interviewer-administered 24-h recalls using the USDA/ARS Automated Multiple-Pass Method [21]. The first recall is administered in person and the second by telephone after a 3-10 day interval. Adults aged ≥19 years who completed at least one 24-h recall that was deemed reliable by the interviewer were included in this study. Using food codes and main food descriptions from the Food and Nutrient Database for Dietary Studies (FNDDS) [22,23,24], fish and shellfish-containing foods were manually identified from all reported food items. For combination foods, such as clam chowder or crab cakes, the amount of fish or shellfish (as a percentage of total recipe weight) was ascertained utilizing recipes from FNDDS. These percentages were multiplied by reported intake to estimate the amount of seafood consumed by each respondent.

#### 2.1.2. Seafood Consumers

Following the dietary recall, survey respondents were asked whether they had eaten any of 18 varieties of fish, 8 varieties of shellfish, breaded fish, or “other” or “unknown” fish or shellfish during the last 30 days. For this study, a person was classified as a consumer of “fish” or “shellfish” if they responded “yes” when asked if they had consumed any type of fish or shellfish, respectively, during the past 30 days. In addition, a person was classified as an “any seafood” consumer if they reported consuming either fish or shellfish or both. 

#### 2.1.3. Socio-Demographic Characteristics

Income categories were created using the ratio of income to poverty, an index calculated by dividing household income by the appropriate US federal poverty threshold [25]. Two income categories were created to differentiate those individuals who live in households eligible for federal food assistance programs such as reduced-price school lunches [26,27] (ratio ≤ 1.85) and those individuals living in households not eligible for such programs (ratio > 1.85). Education level was categorized into three groups: <high school diploma, high school diploma or equivalent, and any post-secondary education. Race and ethnicity were self-reported. In the 2005–2006 survey cycle, Mexican American individuals were oversampled. Beginning in 2007, all Hispanics were oversampled as the numbers of non-Mexican American Hispanic persons in prior surveys were considered too small to produce reliable population estimates for this subgroup [28]. Therefore, when reporting consumption by race-ethnicity, we removed from analysis all individuals (*n* = 2032) who reported any category other than Mexican American, non-Hispanic black, or non-Hispanic white. The final sample size included in this analysis was *n* = 15,407.

### 2.2. Statistical Methods

Logistic regression was used to test whether the percentages of consumers and non-consumers differed by gender, age group, income level, education or race-ethnicity. The effect of each factor was tested separately, using the other factors as covariates. For example, when testing whether the percentages of “seafood consumers” differed between men and women, age group, income level, education and race-ethnicity were included as covariates in the logistic regression model. The SURVEYLOGISTIC procedure in SAS V9.3 (SAS Institute, Inc., Cary, NC, USA) was used for these analyses. Six-year dietary sample weights were included to account for differential probabilities of selection, nonresponse and noncoverage. Bonferroni contrasts were used to compare adjusted percentages within each socio-demographic group. The amounts (g/week) of seafood, fish and shellfish eaten by consumers were estimated using usual intake methodology from the National Cancer Institute (NCI) [29]. Day 1 and day 2 recalls, when available, were used in the analyses to account for the intra-individual variability. The MIXTRAN and DISTRIB V2.1 macros were used to estimate mean intakes and percentiles of the distributions of intakes. For these analyses, 48 balanced repeated replication (BRR) weights calculated using Fay’s adjustment [30] with post stratification, by age groups, gender, and ethnicities, were used instead of the usual day 1 or day 2 recall weights. Covariates included day of week and whether the recall occurred on day 1 or day 2. The two-part form of the usual intake model, which allows the probability of consumption to be correlated with the consumption-day amount, was used because fish and shellfish are episodically consumed foods. Even among consumers, only 33%, 27%, and 18% of respondents reported eating any seafood, fish, or shellfish, respectively, on either day 1 or 2 of their 24-h recalls. Consumption amounts were estimated overall for each sex, age group, income level, education and race-ethnicity category. Consumption amounts among consumers were also calculated separately for men and women for each age group, income level, education and race-ethnicity category. Mean consumption amounts within each socio-demographic group were compared to their corresponding reference group using Z-score testing. 

The recommended weekly intake amount for seafood is based upon energy needs. The most conservative approach is to assume a sedentary activity level to estimate the percentage of individuals meeting weekly intake recommendations, however this likely underestimates actual energy needs for many individuals. The mean estimated energy requirement (EER) [31] assuming sedentary or active activity levels was estimated for gender and age groups using the SURVEYMEANS procedure in SAS V9.3. Average intake of seafood per consumer is presented in ounces to reflect weekly recommendations. Data are reported as percentages or means ± SE. All statistical tests were considered significant at *p* ≤ 0.05.

## 3. Results 

### 3.1. Adults Reporting Any Seafood, Fish, or Shellfish Consumption Over the Past 30 Days

During 2005–2010, a strong majority (83.5 ± 0.5%) of ≥19 years US adults reported eating seafood in the past 30 days; 74.2% ± 0.6% reported eating fish, and 54.2% ± 1.0% reported consuming shellfish (Table 1). Seafood, fish and shellfish consumers varied by socio-demographic characteristics. There was no difference between the percentage of women and men reporting consumption of any seafood (*p* = 0.07) or fish (*p* = 0.28), but more men than women reported eating shellfish (*p* = 0.03). Fewer young adults (aged 19–30 years) than adults aged 31+ years (*p* < 0.0001) reported any seafood or fish consumption and fewer adults aged 71+ years reported consuming shellfish (*p* < 0.0001) than adults 19–70 years. A smaller percentage of individuals from lower-income than from higher-income households reported consuming seafood, fish or shellfish (*p* < 0.0001) and a greater percentage of adults with post-secondary education reported intake of seafood, fish or shellfish in the past 30 days compared to those with a high school diploma or less (*p* < 0.0001). 

There were no differences by race-ethnicity in reported seafood intake (*p* = 0.27); however, more non-Hispanic black adults reported eating fish (*p* = 0.0002) and more Mexican American adults reported eating shellfish (*p* = 0.0001) compared to other race-ethnic groups. Results were similar for both men and women by socio-demographic status when evaluated separately (Table S1: Percentage of women aged ≥19 years who report eating seafood in the past 30 days; Table S2: Percentage of men aged ≥19 years who report eating seafood in the past 30 days).

### 3.2. Reported Intakes of Any Seafood, Fish and Shellfish, by Consumers 

Of the total sample, ~80% reported fish consumption; however, the majority consumed quantities below recommended levels. Thus, it is important to describe amounts consumed only by people who eat seafood. Although the proportion of individuals reporting seafood consumption differed by socio-demographic category, the differences in mean amounts consumed in g/week were small (Table 2). Individuals consumed more fish (128.8 ± 4.9 g/week *vs.* 63.0 ± 4.9 g/week) than shellfish. Men consumed more seafood than women (179.2 ± 9.1 g/week *vs.* 138.6 ± 5.6 g/week, *p* = 0.0001). Men also consumed more fish (142.1 ± 7.7 g/week *vs.* 116.2 ± 5.6 g/week, *p* = 0.01) and shellfish (75.6 ± 7.0 g/week *vs.* 50.4 ± 4.2 g/week, *p* = 0.002) than women. When compared to the youngest age group (19–30 years), adults aged 51–70 years consumed more seafood (172.2 ± 10.5 g/week *vs.* 137.9 ± 9.1 g/week, *p* = 0.01), as did adults aged 31–50 years (161.7 ± 8.4 g/week *vs.* 137.9 ± 9.1 g/week, *p* = 0.05). The intake of adults 71+ years was not different from that of young adults (*p* = 0.62). 

**Table 1:** Percentage of adults aged ≥ 19 years who report eating seafood in the past 30 days ^1^.

Category	*n* ^2^	Any Seafood		Fish		Shellfish	
		% ± SE	*p* value	% ± SE	*p* value	% ± SE	*p* value
All adults	15,407	83.5 ± 0.5		74.2 ± 0.6		54.2 ± 1.0	
Sex			0.075		0.28		0.027
Men	7517	84.2 ± 0.5		74.8 ± 0.7		55.4 ± 1.1 ^a^	
Women	7890	82.7 ± 0.8		73.6 ± 1.0		53.0 ± 1.1 ^b^	
Age (years)			0.0001		0.0001		0.0001
19–30	3592	75.2 ± 1.4 ^a^		60.5 ± 1.7^a^		55.0 ± 1.3 ^a^	
31–50	5340	83.6 ± 0.7 ^b^		72.9 ± 0.9^b^		56.3 ± 1.2 ^a^	
51–70	4742	87.5 ± 0.6 ^c^		81.2 ± 0.7^c^		54.4 ± 1.6 ^a^	
71+	1733	85.3 ± 1.2 ^bc^		81.0 ± 1.3^c^		46.2 ± 1.7 ^b^	
Income ^3,4^			0.0001		0.0001		0.0001
>1.85 poverty threshold	7869	85.9 ± 0.5 ^a^		76.8 ± 0.8^a^		58.6 ± 1.1 ^a^	
0–1.85 poverty threshold	6365	80.8 ± 0.8 ^b^		71.8 ± 0.9^b^		48.6 ± 1.3 ^b^	
Education ^4^			0.0001		0.0001		0.0001
Post-secondary	7265	87.1 ± 0.5 ^a^		80.0 ± 0.6^a^		59.5 ± 1.0 ^a^	
High school or equivalent	3703	81.5 ± 1.0 ^b^		70.8 ± 1.3^b^		49.7 ± 1.5 ^b^	
<High school	4400	78.0 ± 1.1 ^c^		65.8 ± 1.3^c^		49.1 ± 1.7 ^b^	
Race-ethnicity ^5^			0.27		0.0002		0.0001
Non-Hispanic white	7105	82.9 ± 0.8		73.8 ± 0.9^a^		52.6 ± 1.5 ^a^	
Mexican American	3011	83.3 ±1.1		70.2 ± 1.7^a^		60.2 ± 1.4 ^b^	
Non-Hispanic black	3259	85.0 ± 1.0		78.4 ± 1.2^b^		50.6 ± 1.5 ^a^	

^1^ Percentages within categories were estimated using logistic regression and adjusted for all other categories. Percentages within categories not sharing a common letter are significantly different, *p* < 0.05, by Bonferroni contrasts; ^2^ Unweighted sample sizes. Includes only adults whose dietary recalls were deemed to be reliable; ^3^ Income was defined as the ratio of income to poverty; household income divided by federal poverty guidelines; ^4^ Categories do not add up to total due to missing data; ^5^ Categories do not add up to total because “other Hispanic” and “other race” were not included in the analyses.

**Table 2:** Amounts (g/week) of seafood, fish, and shellfish consumed by adults aged ≥ 19 years who report eating seafood in the past 30 days ^1^.

Category	*n* ^2^	Seafood		Fish		Shellfish	
		Mean ± SE (g/week)	*p* value ^3^	Mean ± SE (g/week)	*p* value	Mean ± SE (g/week)	*p* value
All adults	12,506	158.2 ± 5.6		128.8 ± 4.9		63.0 ± 4.9	
Sex							
Men ^4^	6107	179.2 ± 9.1		142.1 ± 7.7		75.6 ± 7.0	
Women	6399	138.6 ± 5.6	0.0001	116.2 ± 5.6	0.01	50.4 ± 4.2	0.002
Age (years)							
19–30 ^4^	2668	137.9 ± 9.1		121.8 ± 9.8		57.4 ± 7.0	
31–50	4402	161.7 ± 8.4	0.05	126.0 ± 7.0	0.74	64.4 ± 7.7	0.50
51–70	4011	172.2 ± 10.5	0.01	140.7 ± 8.4	0.14	65.8 ± 7.7	0.40
71+	1425	144.2 ± 9.8	0.62	114.8 ± 8.4	0.59	60.2 ± 8.4	0.79
Income ^5,6^							
>1.85 poverty threshold ^4^	6721	163.1 ± 7.0		130.2 ± 5.6		64.4 ± 4.9	
0–1.85 poverty threshold	4849	147.0 ± 7.0	0.10	124.6 ± 7.0	0.54	62.3 ± 7.0	0.82
Education ^6^							
Post-secondary ^4^	6285	170.1 ± 7.0		137.9 ± 6.3		61.6 ± 5.6	
High school or equivalent	2931	144.2 ± 9.8	0.03	119.7 ± 10.5	0.14	61.6 ± 8.4	0.97
<High school	3260	137.2 ± 9.8	0.005	108.5 ± 8.4	0.004	70.0 ± 9.1	0.40
Race-ethnicity ^7^							
Non-Hispanic white ^4^	5833	151.2 ± 7.7		123.2 ± 5.6		60.2 ± 1.4	
Mexican American	2290	162.4 ± 11.9	0.41	134.4 ± 12.6	0.42	65.1 ± 4.2	0.23
Non-Hispanic black	2716	167.3 ± 9.8	0.19	139.3 ± 9.1	0.13	60.9 ± 2.1	0.80

^1^ Estimated by MIXTRAN and DISTRIB V2.1 macros using usual intake methodology from the National Cancer institute (NCI); ^2^ Unweighted sample sizes. Includes only adults who reported consuming any seafood in the past 30 days; ^3^ Means within each category were compared to the corresponding reference group using Z test; ^4^ Reference group within category; ^5^ Income was defined as the ratio of income to poverty; household income divided by federal poverty guidelines; ^6^ Categories do not add up to total due to missing data; ^7^ Categories do not add up to total because “other Hispanic” and “other race” were not included in the analyses.

Compared to individuals with higher education, those with a high school degree (144.2 ± 9.8 g/week *vs.* 170.1 ± 7.0 g/week, *p* = 0.03) or lower (137.2 ± 9.8 g/week *vs.* 170.1 ± 7.0 g/week, *p* = 0.005) consumed significantly less of any seafood. Individuals with less than a high school education consumed less fish than individuals with post-secondary education (108.5 ± 8.4 g/week *vs.* 137.9 ± 6.3 g/week, *p* = 0.004). There was no difference by education level in the amount of shellfish consumed. There were no differences in the amounts of any seafood, fish or shellfish consumed by either income or by race-ethnicity categories. These patterns were also similar by socio-demographic group when stratified by sex (Table S3: Amounts (g/week) of seafood, fish, and shellfish consumed by women aged ≥ 19 years who report eating seafood in the past 30 days; Table S4: Amounts (g/week) of seafood, fish, and shellfish consumed by men aged ≥ 19 years who report eating seafood in the past 30 days). Percentiles of intake are presented for all adults (Table S5: Median and percentiles (g/week) of seafood, fish, and shellfish consumed by adults aged ≥ 19 years who report eating any seafood in the past 30 days), women (Table S6: Median and percentiles (g/week) of seafood, fish, and shellfish consumed by women aged ≥ 19 years who report eating any seafood in the past 30 days), and men (Table S7: Median and percentiles (g/week) of seafood, fish, and shellfish consumed by men aged ≥ 19 years who report eating any seafood in the past 30 days).

### 3.3. Proportion of Americans not Meeting Seafood Intake Recommendations 

Comparisons of intakes to the *Dietary Guidelines* recommendation for individuals who reported consuming any seafood are shown in Table 3. It is important to note that the amount recommended varies by energy needs, so while a person with energy needs of 2000 kcal/day (8374 kJ/day) has a recommended intake amount of 8 oz (227 g) seafood/week, a person who requires 3000 kcal/day (12,560 kJ/day) would have a recommended intake of 11 oz (312 g) seafood/wk. Reported mean intake of seafood by all adults 19+ years was 5.6 ± 3.0 oz (158 g/week). With energy needs estimated at a sedentary level, ~80% of all adults did not meet recommendations (range: (78% ± 3%)–(89% ± 2%)). For active adults ~90% did not reach recommendations (range: (82% ± 2%)–(92% ± 2%)). 

**Table 3:** Percentage of Americans not meeting seafood recommendations.

Sex	Age Group	Mean Intake ± SE	Sedentary		Active	
			Recommended Amount	% Below	Recommended Amount	% Below
		oz/week	oz/week	% ± SE	oz/week	% ± SE
Men	19–30	5.5 ± 0.4	11	89 ± 2	11	89 ± 2
	31–50	6.4 ± 0.4	10	81 ± 2	11	84 ± 2
	51–70	6.9 ± 0.4	10	78 ± 3	11	82 ± 2
	71+	5.8 ± 0.4	9	81 ± 3	11	87 ± 2
Women	19–30	4.3 ± 0.4	8	87 ± 3	10	92 ± 2
	31–50	5.0 ± 0.3	8	82 ± 2	10	89 ± 2
	51–70	5.3 ± 0.4	8	80 ± 3	10	87 ± 2
	71+	4.4 ± 0.4	8	86 ± 2	9	89 ± 2

^1^ Calculated using the Estimated Energy Requirement by age and sex group.

## 4. Discussion

In 2005–2010, over three-fourths of US adults reported consuming any seafood in the past 30 days. Of those who ate seafood, most did not consume enough to meet the minimum *Dietary Guidelines* recommendation. Enhancing seafood intake by Americans is a multifaceted issue as seafood is not considered a staple food in many subpopulations in the USA. Addressing underlying factors influencing seafood intake requires an understanding of various interrelated socio-demographic and cultural factors involved. However, the paucity of data regarding socio-demographic characteristics of (1) those who consume seafood compared to those who do not and (2) the quantity and type of seafood eaten by consumers render this topic a difficult issue to explore. 

Our data demonstrate that approximately 80% of US adults eat seafood. The estimates of the proportion of seafood, fish or shellfish consumers in the population are similar to those reported in other recent studies [8,10]. Estimated intake amounts for seafood were approximately 35 g/week higher in this analysis than those reported by Papanikolaou *et al*. (140.7 and 101.5 g/week for men and women, respectively) [10] and by the NCI (138.6 and 99.4 g/week for men and women, respectively) [8]. This discrepancy is likely explained by the fact that we examined the data stratified by consumer while the previous studies reported per capita intake, including both seafood non-consumers and consumers. 

The results indicate that even for Americans who report seafood intake, most are not eating at least the minimum recommended amount. However, this deficiency may be larger than estimated when comparing reported intake to recommendations by energy needs, particularly for men. Assuming a sedentary activity level, our estimated proportion of people who do not meet recommendations is lower than that of a recent NCI report [32] which found that 84% of men and 90% of women did not meet recommended intake amounts, but our results are similar when assuming an active physical activity level. Though simple information in public messaging such a minimum of 8 oz/week is broadly useful, both public health researchers and clinicians need to ensure that recommendations are individualized based on energy needs when considering adequacy of seafood intake. 

### 4.1. Sex and Age

Although there were no differences in the proportions of men and women consuming seafood, men reported consuming greater amount of seafood on a total weight basis. This is not surprising as men have higher overall energy needs and consume, on average, more food than women. When stratified by sex, there were few differences in either proportion of consumers or amount consumed in each socio-demographic category. Thus, segmentation by sex is not necessary and interventions to increase seafood intake would benefit most from a focus on energy needs.

By age group, the lowest prevalence of seafood consumption was reported among the young adults category. There was no difference in amount of seafood consumed in the oldest (71 years and older) and youngest (19–30 years) age groups, but both groups reported lower intake than adults aged 31–70 years. The low intake observed among adults aged 71+ years may reflect lower energy needs. Regarding seafood consumption by young adults, a recent review reported similar results in the United Kingdom, where younger adults consumed less fish than older adults [33]. This relationship may indicate a future decline in seafood consumption if young adults continue their current low intake levels. Researchers in Norway examined the relationship between age and seafood intake [34]. The data suggest that as people age, they are more likely to perceive fish as convenient to prepare, and to consume seafood for perceived health benefits. However, whether the pattern identified in Norway is consistent in other countries and cultures needs validation. Regardless of whether observed differences are due to an aging or a generation effect, further research is needed to identify the underlying reasons for lower seafood consumption among young adults. 

### 4.2. Income and Education

Fewer low-income individuals reported having consumed any seafood, fish, or shellfish in the past 30 days, however, in consumers, the amount eaten did not differ from that of higher-income individuals. These results are in line with previous research with women in the USA showing that as income level increases, the number of times seafood is consumed over 30 days increases [35]. This may indicate that perceived or real cost for some individuals is a barrier to consumption, although it is possible to include high *n*-3 seafood in the diet at minimal cost [36]. Individuals with post-secondary education were more likely to consume any seafood and to report the consumption of more than those with lower education levels. This similarity is most likely due to correlation between education and income in the USA. 

### 4.3. Race-Ethnicity

Our data show no difference in seafood intake by race-ethnicity among US adults. To our knowledge, only two studies have reported seafood intake by race-ethnicity in the USA. Wang *et al*. [12] described trends in amounts of seafood consumption and reported that, in 1999–2004, Non-Hispanic black men and women consumed nearly twice as much seafood as Non-Hispanic whites and Mexican Americans, with little difference between individuals with greater than or less than a high school degree. They showed a slight trend of increasing intake among low, medium, and high income individuals. Mahaffey *et al*. [35] reported that the mean (± SE) number of times seafood was consumed per month was highest among the “other” race-ethnicity category (8.17 ± 1.4), followed by non-Hispanic blacks (5.24 ± 0.3) and non-Hispanic whites (4.43 ± 0.2). Mexican Americans reported the lowest frequency of consumption (2.88 ± 0.1); however, they did not differentiate between fish and shellfish [35]. Direct comparison of our results and these previous studies is confounded by the variety of ways used to describe seafood intake.

### 4.4. Potential Barriers to Seafood Consumption

There is a lack of data defining the barriers that limit seafood consumption in the USA. To date, most studies have focused on perceptions of contaminant risk *vs.* benefits from eating fish, particularly during pregnancy [37,38]. Media messages conveying risk far outnumber those reporting the benefits of eating fish [39], especially farmed fish and seafood [40,41]. In addition to debates about farmed *versus* wild seafood, there are disputes about domestic *versus* foreign, and sustainable *versus* unsustainably produced seafood [42,43]. Even types of aquaculture production systems, such as net-pen enclosures in marine areas *versus* closed recirculating aquaculture systems attract argument and debate. Such competition and debate, while common in developing production sectors, may contribute to a complex message that can deter consumers. Consumers also perceive wild fish to be superior to farmed in taste and in quality [41]. Other studies of consumer choice, mostly from Europe, have identified tradition and habits [44], perceptions of quality (such as with farmed *vs.* wild caught fish) [45], perceived or actual knowledge of health benefits [14,46], and sustainability [47] as important factors that affect the choice to consume seafood. Personal taste preference, price and availability may also be influential factors. The majority of seafood is consumed in restaurants in the USA (nearly 2/3 of the value of seafood sold and consumed is in restaurants) [13], which may suggest that handling and preparation of seafood is challenging for many consumers. Seafood allergies may be another barrier, as 2.5% of Americans report allergies to either fish or shellfish [48]. Formative research is needed to identify barriers to consumption in targeted subgroups of Americans, in particular young adults and those with low income and education. Furthermore, it is likely that factors that limit seafood intake by those who do not consume enough seafood will be different from factors that lead people to not eat seafood. 

Our results confirm that seafood intake in the USA does not meet recommended intake levels. With ~20% of Americans not eating seafood and fewer than 20% of seafood consumers eating recommended amounts, much work remains to move Americans toward seafood consumption at recommended levels. To attain that goal, public health intervention is needed at three levels: first, to shift non-seafood eaters to adoption of regular consumption; second, to increase the amount of seafood consumed; and third, ensure that consumers include n-3 rich seafood in their diets. Adequate production, delivery infrastructure, and waste prevention must be in place to ensure the availability of fresh, affordable seafood to support increased demand if recommendations are to be met. Increasing seafood production for US consumption is not an issue that impacts the US alone, however, and requires acknowledgment of issues that shape seafood consumption worldwide. 70% or more of the seafood the US eats is imported depending on the type of seafood [13]. This import of seafood occurs in a situation in which the majority of wild-catch fisheries are considered fully exploited or overexploited with capture fisheries production having leveled off since 1990 [16]. Meeting food needs has led to an increase in fresh-water aquaculture and marine aquaculture. Recent FAO reports indicate that seafood consumption in Western countries has leveled off or is growing slowly [16]. On the other hand, seafood consumption in Asian and developing countries is growing at a much higher rate through the use of aquacultural practices that require feed and water inputs [16]. In this context, the ability to feed growing populations in addition to meeting seafood consumption recommendations will require coordination of sound aquacultural and fishery practices with trade policy. 

### 4.5. Strengths

Strengths of this study include the use of a large nationally representative sample. Data from three two-year survey cycles were combined to provide statistically reliable estimates for the population subgroups of interest. Two 24-h recalls on non-consecutive days were obtained from a large percentage of the survey respondents, allowing the use of recommended and validated methods for estimating the distribution of usual intakes of episodically-consumed foods. The analytical approaches utilized allowed estimation of the proportion of seafood consumers and for those consumers, the amount usually eaten per day. These estimates are useful for future research in two target areas: adopting seafood consumption and increasing seafood intake in consumers.

### 4.6. Limitations

Limitations of this study include that seafood intake was self-reported and the assumption that those who did not report consuming any seafood in the past 30 days do not consume seafood at all, which potentially under-estimated the proportion of consumers. Furthermore, we did not include the location of participants, so no determination could be made of differences in intake by proximity to fishing areas, which, which, along with cultural tendencies to consume seafood, may impact consumption. However, given the widespread availability of fresh, frozen, and canned seafood in the USA, geographic location likely has less impact on consumption than in previous decades.

We were not able to determine precise quantities of specific species of seafood consumed by socio-demographic category due to small numbers of people reporting eating individual types of seafood in the past 24 h. It is clear that the specific types of seafood eaten by Americans have changed during the survey years analyzed here. For example, the amount of tilapia imported into the USA has doubled from 250 million lbs. in 2004 to 500 million lbs. in 2012 whereas total imports of Atlantic salmon have risen only from 390 to 500 million lbs. during that same period [12]. Such changes in types of seafood consumed can alter subsequent assessment of nutrient intake. For example, tilapia is a low n-3 fish as opposed to Atlantic salmon. Given the health benefits of high n-3 fish consumption, it is important to consider the amount and type of seafood consumed in order to assess its nutrient contribution to the total diet. 

## 5. Conclusions 

In summary, we found that most Americans consume seafood but in inadequate amounts to meet federal dietary guidance, especially when evaluated based upon energy needs. More research is needed to inform interventions to promote adoption and adherence to recommendations, particularly among younger adults and those with low income and education. 

## Supplementary Files

Supplementary File 1
