# Supplementary material for: Intake of Seafood in the US Varies by Age, Income, and Education Level but Not by Race-Ethnicity
Source: Nutrients. 2014 Dec 22;6(12):6060–75. doi: 10.3390/nu6126060 (PMC4277015; doi:10.3390/nu6126060)
Supplement: Supplementary File 1 [file nutrients-06-06060-s001.docx]

Supplementary Information

**Table S1.** Percentage of women aged ≥ 19 years who report eating seafood in the past 30 days ^1^.

| **Category** | ***n* ^2^** | **Any Seafood** | | **Fish** | | **Shellfish** | |
| --- | --- | --- | --- | --- | --- | --- | --- |
|  |  | **% ± SE** | ***p* value** | **% ± SE** | ***p* value** | **% ± SE** | ***p* value** |
| Age (years) |  |  | 0.0001 |  | 0.0001 |  | 0.001 |
| 19–30 | 1929 | 74.0 ± 1.8 ^a^ |  | 59.2 ± 2.2 ^a^ |  | 53.5 ± 1.9 ^a^ |  |
| 31–50 | 2764 | 82.8 ± 1.2 ^b^ |  | 73.6 ± 1.4 ^b^ |  | 54.0 ± 1.6 ^a^ |  |
| 51–70 | 2351 | 87.8 ± 1.0 ^c^ |  | 81.3 ± 1.2 ^c^ |  | 55.3 ± 1.9 ^a^ |  |
| 71+ | 846 | 85.9 ± 1.5 ^bc^ |  | 81.7 ± 1.8 ^c^ |  | 46.7 ± 2.1 ^b^ |  |
| Income ^3,4^ |  |  | 0.0001 |  | 0.0004 |  | 0.0001 |
| >1.85 poverty threshold | 3839 | 85.4 ± 0.8 ^a^ |  | 76.8 ± 1.1 ^a^ |  | 58.0 ± 1.2 ^a^ |  |
| 0–1.85 poverty threshold | 3440 | 80.7 ± 1.1 ^b^ |  | 71.8 ± 1.2 ^b^ |  | 48.5 ± 1.6 ^b^ |  |
| Education ^4^ |  |  | 0.0001 |  | 0.0001 |  | 0.0001 |
| Post-secondary | 3846 | 86.0 ± 0.7 ^a^ |  | 78.9 ± 1.0 ^a^ |  | 57.8 ± 1.2 ^a^ |  |
| High school or equivalent | 1844 | 81.5 ± 1.7 ^b^ |  | 70.7 ± 2.1 ^b^ |  | 49.5 ± 2.0 ^b^ |  |
| <High school | 2176 | 78.2 ± 1.5 ^b^ |  | 67.0 ± 1.4 ^b^ |  | 49.2 ± 2.3 ^b^ |  |
| Race-ethnicity ^5^ |  |  | 0.11 |  | 0.001 |  | 0.026 |
| Non-Hispanic white | 3565 | 82.0 ± 1.1 |  | 72.6 ± 1.2 ^a^ |  | 51.4 ± 1.8 ^a^ |  |
| Mexican American | 1563 | 82.7 ± 1.4 |  | 71.5 ± 2.0 ^a^ |  | 57.8 ± 1.7 ^b^ |  |
| Non-Hispanic black | 1661 | 86.0 ± 1.4 |  | 79.4 ± 1.6^b^ |  | 52.8 ± 1.9 ^ab^ |  |

^1^ Percentages within categories were estimated using logistic regression and adjusted for all other categories. Percentages within categories not sharing a common letter are significantly different, *p* < 0.05, by Bonferroni contrasts; ^2^ Unweighted sample sizes. Includes only adults whose dietary recalls were deemed to be reliable; ^3^ Income was defined as the ratio of income to poverty; household income divided by Federal poverty guidelines; ^4^ Categories do not add up to total due to missing data; ^5^ Categories do not add up to total because “other Hispanic” and “other race” were not included in the analyses.

**Table S2.** Percentage of men aged ≥ 19 years who report eating seafood in the past 30 days ^1^.

| **Category** | ***n* ^2^** | **Any Seafood** | | **Fish** | | **Shellfish** | |
| --- | --- | --- | --- | --- | --- | --- | --- |
|  |  | **% ± SE** | ***p* value** | **% ± SE** | ***p* value** | **% ± SE** | ***p* value** |
| Age (years) |  |  | 0.0001 |  | 0.0001 |  | 0.0001 |
| 19–30 | 1663 | 76.6 ± 1.6 ^a^ |  | 62.0 ± 1.9 ^a^ |  | 55.8 ± 1.8 ^a^ |  |
| 31–50 | 2576 | 84.5 ± 0.9 ^b^ |  | 72.1 ± 1.1 ^b^ |  | 58.5 ± 1.6 ^a^ |  |
| 51–70 | 2391 | 87.1 ± 0.8 ^b^ |  | 81.2 ± 1.0 ^c^ |  | 53.0 ± 2.0 ^a^ |  |
| 71+ | 887 | 84.0 ± 1.4 ^b^ |  | 80.0 ± 1.5 ^c^ |  | 44.9 ± 2.1 ^b^ |  |
| Income ^3,4^ |  |  | 0.0001 |  | 0.0004 |  | 0.0001 |
| >1.85 poverty threshold | 4030 | 86.3 ± 0.6 ^a^ |  | 76.9 ± 0.9 ^a^ |  | 58.9 ± 1.3 ^a^ |  |
| 0–1.85 poverty threshold | 2925 | 80.7 ± 1.0 ^b^ |  | 71.7 ± 1.3 ^b^ |  | 48.1 ± 1.5 ^b^ |  |
| Education ^4^ |  |  | 0.0001 |  | 0.0001 |  | 0.0001 |
| Post-secondary | 3419 | 88.2 ± 0.7 ^a^ |  | 81.2 ± 0.9 ^a^ |  | 61.1 ± 1.5 ^a^ |  |
| High school or equivalent | 1859 | 81.2 ± 1.0 ^b^ |  | 70.7 ± 1.6 ^b^ |  | 49.4 ± 1.8 ^b^ |  |
| <High school | 2224 | 77.7 ± 1.3 ^b^ |  | 64.6 ± 1.9 ^c^ |  | 48.8 ± 2.0 ^b^ |  |
| Race-ethnicity ^5^ |  |  | 0.99 |  | 0.016 |  | 0.0001 |
| Non-Hispanic white | 3540 | 83.8 ± 0.9 |  | 74.8 ± 1.1 ^ab^ |  | 53.5 ± 1.7 ^a^ |  |
| Mexican American | 1448 | 84.0 ± 1.6 |  | 69.3 ± 2.4 ^a^ |  | 62.5 ± 1.9 ^b^ |  |
| Non-Hispanic black | 1598 | 83.7 ± 1.2 |  | 77.1 ± 1.7 ^b^ |  | 47.5 ± 1.9 ^c^ |  |

^1^ Percentages within categories were estimated using logistic regression and adjusted for all other categories. Percentages within categories not sharing a common letter are significantly different, *p* < 0.05, by Bonferroni contrasts; ^2^ Unweighted sample sizes. Includes only adults whose dietary recalls were deemed to be reliable; ^3^ Income was defined as the ratio of income to poverty; household income divided by Federal poverty guidelines; ^4^ Categories do not add up to total due to missing data;
^5^ Categories do not add up to total because “other Hispanic” and “other race” were not included in the analyses.

**Table S3.** Amounts (g/week) of seafood, fish, and shellfish consumed by women aged ≥ 19 years who report eating any seafood in the past 30 days ^1^.

| **Category** | ***n* ^2^** | **Seafood** | | **Fish** | | **Shellfish** | |
| --- | --- | --- | --- | --- | --- | --- | --- |
|  |  | **Mean ± SE (g/week)** | ***p* value ^3^** | **Mean ± SE (g/week)** | ***p* value** | **Mean ± SE (g/week)** | ***p* value** |
| Age (years) |  |  |  |  |  |  |  |
| 19–30 | 1424 | 120.4 ± 9.1 |  | 107.8 ± 9.1 |  | 45.5 ± 4.9 |  |
| 31–50 | 2263 | 141.4 ± 7.7 | 0.07 | 114.1 ± 6.3 | 0.60 | 51.8 ± 6.3 | 0.47 |
| 51–70 | 2008 | 151.2 ± 9.8 | 0.02 | 126.7 ± 8.4 | 0.13 | 53.9 ± 7.0 | 0.34 |
| 71+ | 704 | 126.0 ± 9.1 | 0.66 | 103.6 ± 8.4 | 0.73 | 49.0 ± 7.0 | 0.67 |
| Income ^3,4^ |  |  |  |  |  |  |  |
| >1.85 poverty threshold | 3279 | 142.1 ± 7.0 |  | 116.2 ± 6.3 |  | 51.1 ± 4.2 |  |
| 0–1.85 poverty threshold | 2628 | 129.5 ± 6.3 | 0.19 | 112.7 ± 6.3 | 0.65 | 51.1 ± 6.3 | 0.99 |
| Education ^4^ |  |  |  |  |  |  |  |
| Post-secondary | 3300 | 147.7 ± 7.0 |  | 124.6 ± 6.3 |  | 49.0 ± 4.9 |  |
| High school or equivalent | 1450 | 125.3 ± 9.1 | 0.05 | 108.5 ± 9.8 | 0.17 | 50.4 ± 7.0 | 0.90 |
| <High school | 1629 | 119.0 ± 9.1 | 0.01 | 97.3 ± 7.0 | 0.005 | 57.4 ± 8.4 | 0.38 |
| Race-ethnicity ^5^ |  |  |  |  |  |  |  |
| Non-Hispanic white | 2895 | 131.6 ± 7.0 |  | 110.6 ± 5.6 |  | 48.3 ± 4.2 |  |
| Mexican American | 1200 | 141.4 ± 10.5 | 0.42 | 120.4 ± 11.2 | 0.45 | 53.2 ± 6.3 | 0.51 |
| Non-Hispanic black | 1408 | 149.1 ± 9.1 | 0.13 | 127.4 ± 8.4 | 0.11 | 51.1 ± 5.6 | 0.70 |

^1^ Estimated by MIXTRAN and DISTRIB V2.1 macros using usual intake methodology from the National Cancer institute (NCI); ^2^ Unweighted sample sizes. Includes only adults who reported consuming any seafood in the past 30 days; ^3^ Means within each category were compared to the corresponding reference group using Z test;
^4^ Reference group within category; ^5^ Income was defined as the ratio of income to poverty; household income divided by Federal poverty guidelines; ^6^ Categories do not add up to total due to missing data; ^7^ Categories do not add up to total because “other Hispanic” and “other race” were not included in the analyses.

**Table S4.** Amounts (g/week) of seafood, fish, and shellfish consumed by men aged ≥ 19 years who report eating any seafood in the past 30 days ^1^.

| **Category** | ***n* ^2^** | **Seafood** | | **Fish** | | **Shellfish** | |
| --- | --- | --- | --- | --- | --- | --- | --- |
|  |  | **Mean ± SE (g/week)** | ***p* value ^3^** | **Mean ± SE (g/week)** | ***p* value** | **Mean± SE (g/week)** | ***p* value** |
| Age (years) |  |  |  |  |  |  |  |
| 19–30 | 1244 | 156.1 ± 11.9 |  | 132.3 ± 11.2 |  | 68.6 ± 9.8 |  |
| 31–50 | 2139 | 182.7 ± 11.9 | 0.11 | 139.3 ± 9.8 | 0.63 | 77.0 ± 9.8 | 0.53 |
| 51–70 | 2003 | 195.3 ± 12.6 | 0.02 | 155.4 ± 11.2 | 0.14 | 80.5 ± 9.1 | 0.37 |
| 71+ | 721 | 163.8 ± 11.9 | 0.64 | 127.4 ± 9.8 | 0.75 | 74.2 ± 10.5 | 0.69 |
| Income ^3,4^ |  |  |  |  |  |  |  |
| >1.85 poverty threshold | 3442 | 184.1 ± 9.8 |  | 143.5 ± 8.4 |  | 77.0 ± 7.7 |  |
| 0–1.85 poverty threshold | 2221 | 168.0 ± 10.5 | 0.26 | 137.9 ± 9.8 | 0.67 | 77.0 ± 9.8 | 1.00 |
| Education ^4^ |  |  |  |  |  |  |  |
| Post-secondary | 2985 | 189.7 ± 9.8 |  | 151.9 ± 8.4 |  | 74.2 ± 8.4 |  |
| High school or equivalent | 1481 | 161.7 ± 12.6 | 0.07 | 130.2 ± 12.6 | 0.15 | 75.6 ± 10.5 | 0.91 |
| <High school | 1631 | 152.6 ± 12.6 | 0.017 | 117.6 ± 10.5 | 0.01 | 86.8 ± 11.2 | 0.35 |
| Race-ethnicity ^5^ |  |  |  |  |  |  |  |
| Non-Hispanic white | 2938 | 170.1 ± 10.5 |  | 134.4 ± 8.4 |  | 72.1 ± 7.7 |  |
| Mexican American | 1090 | 180.6 ± 15.4 | 0.59 | 145.6 ± 16.1 | 0.53 | 79.1 ± 11.2 | 0.63 |
| Non-Hispanic black | 1308 | 193.2 ± 14.0 | 0.19 | 156.1 ± 12.6 | 0.15 | 77.0 ± 8.4 | 0.71 |

^1^ Estimated by MIXTRAN and DISTRIB V2.1 macros using usual intake methodology from the National Cancer institute (NCI); ^2^ Unweighted sample sizes. Includes only adults who reported consuming any seafood in the past 30 days; ^3^ Means within each category were compared to the corresponding reference group using Z test;
^4^ Reference group within category; ^5^ Income was defined as the ratio of income to poverty; household income divided by Federal poverty guidelines; ^6^ Categories do not add up to total due to missing data; ^7^ Categories do not add up to total because “other Hispanic” and “other race” were not included in the analyses.

**Table S5.** Median and percentiles (g/week) of seafood, fish, and shellfish consumed by adults aged ≥ 19 years who report eating any seafood in the past 30 days ^1,2^.

| **Category** | **Seafood** | | | | | | | **Fish** | | | | | | | **Shellfish** | | | | | | |
| --- | --- | --- | --- | --- | --- | --- | --- | --- | --- | --- | --- | --- | --- | --- | --- | --- | --- | --- | --- | --- | --- |
|  | ***Percentiles*** | | | | | | | ***Percentiles*** | | | | | | | ***Percentiles*** | | | | | | |
|  | **5** | **10** | **25** | **50** | **75** | **90** | **95** | **5** | **10** | **25** | **50** | **75** | **90** | **95** | **5** | **10** | **25** | **50** | **75** | **90** | **95** |
| All adults | 25.0 | 35.8 | 64.2 | 119.0 | 210.5 | 333.4 | 426.4 | 27.6 | 37.3 | 61.0 | 103.3 | 168.6 | 253.5 | 317.0 | 11.5 | 15.9 | 27.2 | 48.0 | 82.7 | 130.2 | 166.7 |
| Sex |  |  |  |  |  |  |  |  |  |  |  |  |  |  |  |  |  |  |  |  |  |
| Men | 29.1 | 41.5 | 73.9 | 136.2 | 239.2 | 375.6 | 475.0 | 30.7 | 41.5 | 67.8 | 114.2 | 186.5 | 278.8 | 348.0 | 14.8 | 20.3 | 34.0 | 59.2 | 99.9 | 153.6 | 193.3 |
| Women | 22.2 | 31.8 | 56.9 | 104.9 | 184.5 | 290.2 | 370.6 | 25.3 | 34.2 | 55.8 | 93.9 | 152.4 | 227.2 | 282.9 | 9.7 | 13.4 | 22.4 | 39.3 | 66.7 | 103.3 | 131.6 |
| Age (years) |  |  |  |  |  |  |  |  |  |  |  |  |  |  |  |  |  |  |  |  |  |
| 19–30 | 20.2 | 29.1 | 53.4 | 100.7 | 182.0 | 293.8 | 380.1 | 24.6 | 33.5 | 55.6 | 95.8 | 159.4 | 243.4 | 308.6 | 10.9 | 14.9 | 25.1 | 44.0 | 75.2 | 117.5 | 149.7 |
| 31–50 | 24.5 | 35.4 | 64.2 | 120.4 | 214.8 | 343.4 | 440.9 | 25.6 | 35.0 | 58.1 | 99.2 | 165.1 | 251.2 | 317.7 | 12.4 | 16.9 | 28.5 | 49.8 | 84.8 | 131.1 | 166.7 |
| 51–70 | 27.0 | 39.1 | 70.3 | 130.3 | 229.4 | 360.6 | 458.9 | 29.2 | 40.0 | 65.9 | 112.6 | 184.7 | 278.0 | 347.6 | 13.0 | 17.7 | 29.7 | 51.6 | 87.0 | 133.6 | 168.8 |
| 71+ | 21.6 | 31.1 | 56.7 | 106.6 | 190.8 | 307.0 | 396.5 | 23.5 | 31.9 | 53.0 | 90.7 | 150.9 | 229.9 | 289.4 | 11.6 | 15.8 | 26.6 | 46.5 | 79.0 | 122.9 | 156.8 |
| Income ^3^ |  |  |  |  |  |  |  |  |  |  |  |  |  |  |  |  |  |  |  |  |  |
| >1.85 poverty threshold | 24.6 | 35.4 | 64.6 | 121.5 | 216.7 | 345.9 | 445.1 | 26.9 | 36.6 | 60.5 | 103.3 | 171.0 | 259.1 | 325.2 | 13.2 | 17.9 | 29.5 | 50.6 | 84.1 | 128.3 | 162.2 |
| 0–1.85 poverty threshold | 21.1 | 30.6 | 56.4 | 107.2 | 194.0 | 314.1 | 408.7 | 25.4 | 34.7 | 57.1 | 97.9 | 162.9 | 249.5 | 315.6 | 12.5 | 16.9 | 28.1 | 48.5 | 81.4 | 125.4 | 158.4 |
| Education |  |  |  |  |  |  |  |  |  |  |  |  |  |  |  |  |  |  |  |  |  |
| Post-secondary | 26.8 | 38.7 | 69.6 | 129.0 | 226.5 | 356.2 | 455.3 | 28.9 | 39.4 | 64.8 | 110.2 | 181.2 | 271.6 | 340.8 | 11.8 | 16.1 | 27.0 | 47.4 | 80.6 | 125.0 | 159.1 |
| High school or equivalent | 21.3 | 30.8 | 56.3 | 105.9 | 190.7 | 308.6 | 399.3 | 23.9 | 32.6 | 54.1 | 93.5 | 156.8 | 240.9 | 305.9 | 11.9 | 16.2 | 27.3 | 47.7 | 80.9 | 126.2 | 160.7 |
| <High school | 19.8 | 28.6 | 52.3 | 99.6 | 180.5 | 294.7 | 385.5 | 21.4 | 29.2 | 48.8 | 84.1 | 141.3 | 218.1 | 277.6 | 13.1 | 18.1 | 30.6 | 53.8 | 92.4 | 144.1 | 183.5 |
| Race-ethnicity ^4^ |  |  |  |  |  |  |  |  |  |  |  |  |  |  |  |  |  |  |  |  |  |
| Non-Hispanic white | 25.5 | 35.9 | 62.9 | 114.5 | 198.8 | 312.8 | 399.5 | 26.0 | 35.1 | 57.5 | 97.8 | 161.2 | 244.8 | 307.7 | 14.3 | 18.8 | 29.8 | 48.9 | 78.7 | 116.8 | 145.6 |
| Mexican American | 27.0 | 38.3 | 67.2 | 122.2 | 213.9 | 337.7 | 431.6 | 28.3 | 37.9 | 62.4 | 106.5 | 176.2 | 266.9 | 335.3 | 15.4 | 20.4 | 32.1 | 52.9 | 85.1 | 127.1 | 159.0 |
| Non-Hispanic black | 29.5 | 41.5 | 72.0 | 129.0 | 221.1 | 342.0 | 433.8 | 30.3 | 41.0 | 66.6 | 112.4 | 182.5 | 272.6 | 341.0 | 14.6 | 19.4 | 30.6 | 49.8 | 79.7 | 117.3 | 146.0 |

^1^ Estimated by MIXTRAN and DISTRIB V2.1 macros using usual intake methodology from the National Cancer institute (NCI); ^2^ Includes only adults who reported consuming any seafood in the past 30 days;
^3^ Income was defined as the ratio of income to poverty; household income divided by Federal poverty guidelines; ^4^ “Other Hispanic” and “other race” were not included in the analyses.

**Table S6.** Median and percentiles (g/week) of seafood, fish, and shellfish consumed by women aged ≥ 19 years who report eating any seafood in the past 30 days ^1,2^

| **Category** | **Seafood** | | | | | | | **Fish** | | | | | | | **Shellfish** | | | | | | |
| --- | --- | --- | --- | --- | --- | --- | --- | --- | --- | --- | --- | --- | --- | --- | --- | --- | --- | --- | --- | --- | --- |
|  | ***Percentiles*** | | | | | | | ***Percentiles*** | | | | | | | ***Percentiles*** | | | | | | |
|  | **5** | **10** | **25** | **50** | **75** | **90** | **95** | **5** | **10** | **25** | **50** | **75** | **90** | **95** | **5** | **10** | **25** | **50** | **75** | **90** | **95** |
| Age (years) |  |  |  |  |  |  |  |  |  |  |  |  |  |  |  |  |  |  |  |  |  |
| 19–30 | 18.6 | 26.7 | 48.1 | 90.0 | 159.9 | 254.0 | 326.9 | 23.3 | 31.5 | 51.4 | 86.7 | 141.7 | 211.8 | 265.1 | 8.8 | 11.9 | 20.1 | 35.1 | 60.0 | 93.5 | 120.1 |
| 31–50 | 22.7 | 32.4 | 57.9 | 106.8 | 188.4 | 295.2 | 377.4 | 24.9 | 33.4 | 54.4 | 91.8 | 149.6 | 223.9 | 278.8 | 9.9 | 13.5 | 22.7 | 39.8 | 67.6 | 105.0 | 135.0 |
| 51–70 | 24.9 | 35.6 | 63.6 | 115.9 | 202.4 | 313.2 | 396.6 | 28.3 | 37.9 | 62.0 | 103.6 | 166.5 | 246.4 | 303.6 | 10.7 | 14.6 | 24.2 | 42.1 | 71.1 | 108.8 | 137.6 |
| 71+ | 20.1 | 28.3 | 51.1 | 95.0 | 167.1 | 263.6 | 339.0 | 23.0 | 30.7 | 50.2 | 83.9 | 135.8 | 201.9 | 251.8 | 9.4 | 12.8 | 21.9 | 38.0 | 64.5 | 100.8 | 128.0 |
| Income ^3^ |  |  |  |  |  |  |  |  |  |  |  |  |  |  |  |  |  |  |  |  |  |
| >1.85 poverty threshold | 22.5 | 32.3 | 58.2 | 108.1 | 189.6 | 297.3 | 379.4 | 26.0 | 35.2 | 56.8 | 94.8 | 153.2 | 226.9 | 280.6 | 10.4 | 14.1 | 23.5 | 40.1 | 66.8 | 102.5 | 129.6 |
| 0–1.85 poverty threshold | 19.5 | 28.3 | 51.0 | 96.1 | 171.2 | 275.4 | 355.6 | 24.9 | 33.3 | 53.7 | 90.4 | 147.7 | 220.4 | 275.2 | 10.3 | 14.0 | 23.1 | 39.7 | 66.6 | 102.9 | 131.0 |
| Education |  |  |  |  |  |  |  |  |  |  |  |  |  |  |  |  |  |  |  |  |  |
| Post-secondary | 25.8 | 36.3 | 63.6 | 114.3 | 195.1 | 302.9 | 383.7 | 7.2 | 37.9 | 61.3 | 101.9 | 163.0 | 241.1 | 297.5 | 9.3 | 12.8 | 21.5 | 37.7 | 64.5 | 100.3 | 127.7 |
| High school or equivalent | 19.0 | 27.0 | 48.2 | 88.1 | 155.6 | 249.6 | 324.7 | 21.0 | 28.1 | 45.8 | 76.9 | 126.9 | 193.6 | 243.1 | 10.9 | 14.9 | 25.2 | 44.2 | 75.4 | 117.3 | 150.1 |
| <High school | 20.7 | 29.2 | 51.7 | 94.2 | 164.0 | 261.0 | 336.7 | 23.7 | 31.6 | 51.3 | 86.5 | 141.5 | 214.2 | 267.9 | 9.5 | 13.1 | 22.1 | 38.8 | 66.0 | 102.2 | 129.6 |
| Race-ethnicity ^4^ |  |  |  |  |  |  |  |  |  |  |  |  |  |  |  |  |  |  |  |  |  |
| Non- Hispanic white | 23.2 | 32.3 | 56.3 | 100.9 | 173.6 | 271.4 | 345.5 | 24.7 | 33.1 | 53.5 | 89.5 | 144.9 | 216.7 | 271.0 | 11.6 | 15.3 | 23.9 | 39.2 | 62.9 | 103.1 | 129.1 |
| Mexican American | 24.7 | 34.4 | 60.1 | 108.0 | 186.0 | 292.8 | 375.3 | 26.7 | 35.7 | 57.8 | 97.5 | 157.4 | 235.8 | 291.9 | 12.6 | 16.6 | 26.3 | 43.3 | 69.4 | 103.1 | 129.1 |
| Non-Hispanic black | 27.3 | 38.4 | 66.2 | 117.0 | 197.4 | 301.4 | 378.0 | 29.4 | 39.2 | 62.9 | 104.4 | 166.5 | 245.8 | 304.5 | 11.6 | 15.3 | 23.9 | 39.2 | 62.9 | 93.7 | 116.8 |

^1^ Estimated by MIXTRAN and DISTRIB V2.1 macros using usual intake methodology from the National Cancer institute (NCI); ^2^ Includes only adults who reported consuming any seafood in the past 30 days;
^3^ Income was defined as the ratio of income to poverty; household income divided by Federal poverty guidelines; ^4^ “Other Hispanic” and “other race” were not included in the analyses.

**Table S7.** Median and percentiles (g/week) of seafood, fish, and shellfish consumed by men aged ≥ 19 years who report eating any seafood in the past 30 days ^1,2^.

| **Category** | **Seafood** | | | | | | | **Fish** | | | | | | | **Shellfish** | | | | | | |
| --- | --- | --- | --- | --- | --- | --- | --- | --- | --- | --- | --- | --- | --- | --- | --- | --- | --- | --- | --- | --- | --- |
|  | ***Percentiles*** | | | | | | | ***Percentiles*** | | | | | | | ***Percentiles*** | | | | | | |
|  | **5** | **10** | **25** | **50** | **75** | **90** | **95** | **5** | **10** | **25** | **50** | **75** | **90** | **95** | **5** | **10** | **25** | **50** | **75** | **90** | **95** |
| Age (years) |  |  |  |  |  |  |  |  |  |  |  |  |  |  |  |  |  |  |  |  |  |
| 19–30 | 24.4 | 35.1 | 63.0 | 116.1 | 206.2 | 328.9 | 420.4 | 28.4 | 38.4 | 62.4 | 105.6 | 172.8 | 261.3 | 328.4 | 13.2 | 18.2 | 30.2 | 52.9 | 90.2 | 138.8 | 177.5 |
| 31–50 | 29.5 | 42.2 | 75.5 | 138.8 | 243.3 | 381.6 | 485.7 | 30.2 | 40.8 | 66.6 | 112.4 | 182.9 | 273.8 | 342.1 | 15.2 | 20.7 | 34.7 | 60.3 | 101.8 | 156.9 | 197.5 |
| 51–70 | 33.3 | 47.1 | 83.7 | 151.8 | 260.1 | 403.3 | 510.0 | 34.8 | 46.6 | 76.0 | 126.8 | 205.8 | 303.5 | 374.9 | 16.2 | 21.9 | 36.7 | 63.4 | 106.2 | 163.0 | 205.5 |
| 71+ | 26.3 | 37.7 | 67.3 | 124.6 | 217.2 | 342.3 | 440.2 | 27.7 | 37.2 | 60.9 | 103.0 | 167.4 | 251.2 | 313.2 | 14.4 | 19.6 | 33.1 | 58.0 | 97.7 | 151.3 | 192.0 |
| Income ^3^ |  |  |  |  |  |  |  |  |  |  |  |  |  |  |  |  |  |  |  |  |  |
| >1.85 poverty threshold | 29.5 | 42.3 | 76.0 | 140.4 | 246.5 | 384.2 | 487.3 | 32.1 | 43.2 | 69.9 | 116.3 | 188.2 | 280.4 | 349.7 | 16.0 | 21.6 | 35.7 | 61.1 | 101.2 | 153.3 | 192.7 |
| 0–1.85 poverty threshold | 25.8 | 37.0 | 66.4 | 124.5 | 221.8 | 356.7 | 460.0 | 30.2 | 41.2 | 66.4 | 111.3 | 181.4 | 270.8 | 337.6 | 15.8 | 21.4 | 35.4 | 60.4 | 101.0 | 154.1 | 193.4 |
| Education |  |  |  |  |  |  |  |  |  |  |  |  |  |  |  |  |  |  |  |  |  |
| Post-secondary | 33.9 | 47.5 | 82.5 | 147.6 | 252.5 | 388.0 | 490.0 | 34.5 | 46.2 | 74.8 | 124.0 | 199.4 | 295.2 | 365.3 | 14.1 | 19.5 | 33.0 | 57.8 | 97.4 | 150.6 | 190.1 |
| High school or equivalent | 25.0 | 35.6 | 62.5 | 113.9 | 201.0 | 319.1 | 412.6 | 25.0 | 33.4 | 54.5 | 92.3 | 152.8 | 233.5 | 296.7 | 16.9 | 23.0 | 38.6 | 67.1 | 114.5 | 177.7 | 226.5 |
| <High school | 27.2 | 38.6 | 67.1 | 121.7 | 212.6 | 335.9 | 432.1 | 27.9 | 38.0 | 61.9 | 104.1 | 170.0 | 258.0 | 325.2 | 14.5 | 20.1 | 33.7 | 58.6 | 98.8 | 153.9 | 196.2 |
| Race-ethnicity ^4^ |  |  |  |  |  |  |  |  |  |  |  |  |  |  |  |  |  |  |  |  |  |
| Non-Hispanic white | 30.4 | 42.6 | 73.6 | 131.7 | 224.9 | 347.2 | 441.8 | 30.0 | 40.2 | 64.9 | 108.7 | 176.1 | 263.3 | 327.7 | 17.4 | 22.8 | 36.1 | 59.1 | 94.5 | 139.7 | 173.3 |
| Mexican American | 32.1 | 44.7 | 77.8 | 138.5 | 237.9 | 368.5 | 473.1 | 32.4 | 43.9 | 70.4 | 116.9 | 190.7 | 284.6 | 358.8 | 18.8 | 24.7 | 39.1 | 63.9 | 103.3 | 153.2 | 190.9 |
| Non-Hispanic black | 35.9 | 50.3 | 86.3 | 151.7 | 256.7 | 388.6 | 487.8 | 36.5 | 48.0 | 77.2 | 127.4 | 203.9 | 301.7 | 377.0 | 18.8 | 24.8 | 38.9 | 63.3 | 99.9 | 146.6 | 181.0 |

^1^ Estimated by MIXTRAN and DISTRIB V2.1 macros using usual intake methodology from the National Cancer institute (NCI); ^2^ Includes only adults who reported consuming any seafood in the past 30 days;
^3^ Income was defined as the ratio of income to poverty; household income divided by Federal poverty guidelines; ^4^ “Other Hispanic” and “other race” were not included in the analyses.

© 2014 by the authors; licensee MDPI, Basel, Switzerland. This article is an open access article distributed under the terms and conditions of the Creative Commons Attribution license (http://creativecommons.org/licenses/by/4.0/).
